# The Environmental Footprint of Neurosurgery Operations: An Assessment of Waste Streams and the Carbon Footprint

**DOI:** 10.3390/ijerph19105995

**Published:** 2022-05-15

**Authors:** Sayed Samed Talibi, Teresa Scott, Rahim A. Hussain

**Affiliations:** Department of Neurosurgery, University Hospital Coventry and Warwickshire, Coventry CV2 2DX, UK; teresa.scott@doctors.org.uk (T.S.); rahim.hussain@uhcw.nhs.uk (R.A.H.)

**Keywords:** biomedical waste management, carbon footprint, environmental sustainability, hospital ecomanagement, neurosurgery, surgery sustainability, surgical system improvement

## Abstract

Healthcare in England generates 24.9 million tonnes of carbon dioxide equivalents (CO_2_e), equating to approximately 4% of the total national output of greenhouse gases (GHG), and of this, 10% is from the manufacturing of medical equipment. Operating theatres are a major contributor of biomedical waste, especially consumables, and are three-to-six times more energy intensive than the rest of the hospital. This study seeks to quantify and evaluate the carbon cost, or footprint, of neurosurgery at a single institution in England. A single neurosurgical operation generates, on average, 8.91 kg of waste per case, equivalent to 24.5 CO_2_e kg per case, mostly from single-use equipment. Per annum, 1300 neurosurgical operative cases are performed with total waste generation of 11,584.4 kg/year and a carbon footprint of 31,859 (kg) CO_2_e. The challenge of achieving net zero GHG presents an opportunity to catalyse innovation and sustainability in neurosurgery, from how care is delivered, through to equipment use and surgical methodologies. This should improve the quality of healthcare provision to patients and yield potential cost savings.

## 1. Introduction

In 2015, the United Nations member states adopted 17 sustainable development goals, a prelude to the UK Prime Minister’s announcement in late 2020 of the pathways to net zero greenhouse gas emissions (GHG) [1,2]. Any healthcare system should be cognisant that its mission and vision should align to environmental, social, and governance (ESG) principles that have been adopted by most large organisations. The NHS has been driving towards a path of sustainable healthcare [3,4].

The National Health Service (NHS) is estimated to generate 24.9 million metric tons of CO_2_ or approximately 4.4% of all greenhouse gases in England [4,5]. The provision of first-rate healthcare is complicated by the resource intensiveness, and, accordingly, this equates to 25% of public sector emissions [6,7]. Hospitals, as health producers, are polluting; the moral imperative of doing no harm, i.e., “primun non nocere”, should be holistic in its application, taking account of risks to the external environment [8]. The steps involved in any patient healthcare journey including neurosurgery generate vast quantities of biomedical waste and CO_2_ [9,10]. Operating theatres generate 21–30% of hospital waste and are three-to-six times more energy intensive than the rest of the hospital [7,11]. The everyday activities in a hospital are energy intensive, including the use of expensive surgical equipment, sterilisation processes, advanced operative technologies, and disposable equipment [12,13]. It has, for example, been shown that 13% of the disposable items opened for neurosurgical procedures are discarded without use [9]. The climate impacts of healthcare are largely in countries with developed economies and are considered as a necessary cost to the delivery of quality care [13,14].

This study seeks to quantify and evaluate the carbon cost, or footprint, of neurosurgery at a single institution in England. Quantification of the environmental impact should assist in determining the potential value of mitigation and harm, thereby identifying steps or measures to help formulate recommendations to minimise the carbon impact of neurosurgery and, at the macro-level, the global climate. The choice of topic reflects the experiences of the authors, all working within neurosurgery in a single institution in England, in addition to the paucity of studies on this topic, as much of the literature focuses on the other surgical specialities [11]. The wider aim of this paper is to stimulate thought and research into the impact of anthropogenic factors on the quality of the environment through the prism of healthcare provision.

## 2. Materials and Methods

The healthcare facility known as University Hospitals Coventry and Warwickshire NHS trust (UHCW) was recently constructed as part of the NHS’s redevelopment of its century-old infrastructure, with funding derived from public finance initiative contracts, partnering with the private sector. The site is an academic hospital and major trauma centre with over 1200 beds, a full complement of specialist services, and a regional centre for neurosurgical practice based in England.

The construction and design of the hospital’s theatre are typical of most other British institutions, with construction of a separate anaesthetic induction room for each operative suite. This process helps to improve the flow in combination with other structural and process differences, resulting in higher throughput. It has two dedicated neurosurgical theatres plus additional out-of-hours theatres for emergency cases.

Data were collected from 200 adult neurosurgical cases at the institution over a one-year period (January to December 2021). The volumes of biomedical waste were estimated from a representative sample using audits rather than having access to all the actual annual data for each stream. The cases were not consecutive but rather a reflection of all cases by a single observer with no case overlap, i.e., a single neurosurgical doctor in training or resident and not a consultant. At UHCW, 1300 operative cases varying from simple injections to complex microsurgery from an estimated total of 39,372 operations were performed by the end of 2021 [15]. The period of assessment did coincide with the latter period of the COVID-19 pandemic, although, reviewing the number of neurosurgical cases operated, this only reflected a 5% decrease in caseload.

The weight of the generated waste was recorded for each class of operative case. The disposable items that were opened but not used, i.e., discarded, were also recorded and included as part of the general waste count. The generated waste was catalogued for each class of surgical intervention, i.e., cranial complex and routine, spinal complex and routine, and others, then extrapolated for similar types of operative cases. The actual observation of the waste collected accounts for 20% of the total 200 cases in the study. For each type of waste, its disposal was investigated through local queries, hospital standard operating procedures, and discussions with theatre management. The surgical waste was segregated into the following streams: cytotoxic waste; domestic waste; fluid waste; hazardous waste; municipal solid waste; recycling; reusable textiles; and sharps.

Municipal waste is a separate stream of waste from the corridors outside the operating theatres, and is classed as non-contaminated and non-infectious, so that no other waste type should enter it. Recycling is collected by local vendors. Laundry is processed on site, and the production emissions were omitted given that the textiles are reusable.

The DEFRA online tool for greenhouse gas (GHG) life-cycle emissions was utilised when applying the conversion factors for waste disposal generated throughout the supply chain [16]. An estimation was made of the emissions of various material waste streams: glass (895 kg CO_2_e per tonne); plastics (3179 kg CO_2_e per tonne); and steel (2708 kg CO_2_e per tonne).

This study excludes the impact of complex spinal instrumentation used for fixations, emissions from the manufactory of shunt components, and titanium cranial implants. The reprocessing of surgical instruments and third-party single-use device are excluded, as well as the human resources required, i.e., personal/private transportation of the staff members to and from work. The energy demands of the facility, e.g., heating and lighting, are beyond the scope of this paper.

## 3. Results

In a one-year period, the department of neurosurgery will undertake approximately 1300 operative cases, varying from simple injections to complex microsurgery, from an estimated total of 39,372 operations hospital wide [15]. This would account for 3.3% of all operations at UHCW. If extrapolated from the average of 8.91 kg of waste generated per case, equivalent to 24.5 CO_2_e kg per case, for 1300 neurosurgical cases, the resulting total is 11,584.4 kg/year of waste and a carbon footprint of 31,859 (kg) CO_2_e. Table 1 features a breakdown of the waste according to the type of neurosurgery. Table 2 is a breakdown of the waste according to the types cross-referenced with the DEFRA tool.

## 4. Discussion

Over the last decade, the healthcare system has been aware of its carbon footprint, with estimates of the biomedical waste generation and CO_2_ output in the public domain. It has filtered down to each individual trust to demonstrate commitments to the WHO’s 17 sustainable development goals, and the COP 26 pact reinforces a tectonic push to reduce the environmental impact of organisations that have been, in some way, exempt [2,17]. There is existing evidence on the environmental impact of surgical activities, and this encompasses individual products, procedures, or comparisons of alterative procedures [5,10,11,18]. This study examines the carbon burden associated with neurosurgical interventions and offers an insight into the environmental costs of surgical training in the UK at a single institution.

The UK has one of the longest surgical training programmes, particularly for neurosurgery, other non-surgical commitments during training notwithstanding. While it is outside the scope of this paper to make a direct comparison with operations performed by a consultant, it could be suggested that trainees are less efficient in the utilisation of resources, e.g., adverse bleeding during an operation with associated usage of more single-use surgical swabs.

The surgical theatre is a multi-disciplinary space, and would, therefore, be an ideal forum to bring about effective change. The surgical operating theatre is a physically defined area under the control of non-clinical managers and has its own discreet financial stream; furthermore, it is responsible for its own supply chain for equipment procurement. The multi-professional team occupying the operating theatres could tangibly push to reduce the carbon footprint through the critical assessment of patterns of behaviour. The operating theatre is the most resource-intensive sector in most hospitals and any reduction in greenhouse gas emissions will be high yield. The operating theatres are major contributors of waste, especially consumables generating 21–30% of hospital waste and are three-to-six times more energy intensive than the rest of the hospital [7,11].

The hospital’s own published data, up until the COVID-19 pandemic, shows that 3065 tonnes of waste per annum is generated, of which up to 47% is recycled, with equivalent emissions of 66.69 tonnes of CO_2_e (see Appendix A Table A1) [15,19,20]. The hospital has taken steps under the provision of the Public Services (Social Value) Act 2012, establishing a Procurement Working Group responsible for analysing all aspects of sustainable procurement across the Trust [19].

Neurosurgery generates the equivalent of 24.5 kg CO_2_e per case, in comparison to the UK average of 173 kg CO_2_e per case, a relatively small fraction [11,21]. The complexity of neurosurgical interventions and mitigation against the risks of variant Creutzfeldt-Jakob disease (CJD) spreading through cerebrospinal fluid contribute to a relatively high wastage rate, as 13% of disposable items opened for neurosurgical procedures are discarded without use [9]. It should be considered that the quality of evidence for this association is rated as very low and there is no clear association of CJD with neurosurgery, similarly for ophthalmic surgery [22]. The 24.5 kg CO_2_e per neurosurgical case is further overshadowed by cataract surgery, with emissions of 182 kg CO2e per operation, and surgically treated gastro-oesophageal reflux disease (GORD) at 1375 kg CO_2_e per fraction [11,21]. The same intended surgical operation can also vary depending on the geography; the same cataract surgery, e.g., in India is estimated to expend 6 kg CO2e per case [11]. The availability of resources and materials reflects the often-lower carbon expenditure in developing healthcare systems, where equipment is often recycled and reusable.

The WHO’s report and guidelines on Surgical Site Infection Prevention adjudicates that there is a low quality of evidence for the conclusion that disposable single-use drapes and gowns are neither beneficial nor harmful in reducing the SSI rate when compared to reusable drapes and gowns [18]. It also notes that the resource implications suggest that disposable and reusable surgical drapes and gowns are probably similar in cost, although there is no indication of the volumes of waste generated nor the subsequent carbon footprint. The COVID-19 pandemic and fear of litigation, in addition to aggressive promotion by the manufacturing companies, promote the use of disposable drapes and gowns, an acceptable norm in the operating theatre environment.

The move to cheaper disposable equipment, due to the rising costs and infection control, has also led to an increase in the use of disposable surgical instruments in daily hospital practices. An example to note is the use of operative scissors, where a single-use equivalent can cost up to GBP 4.26 and expend 835 gCO_2_/e per use, with reusable scissors costing a fraction at GBP 1.42 and expending 64 gCO_2_/e per use [23,24]. It should be considered that the costs related to the sterilisation of the reusable product are case-specific and can reduce the economic benefit of the reusable scissors to zero.

The post-COVID-19 recovery of the NHS has left over five million people on the waiting list for surgery [20,25]. The need for surgery, however, pre-dates the pandemic, with the increase in the burden of surgical disease expected to involve 143 million additional surgical procedures each year by 2030, to save lives and prevent disability [26]. This push must be reconciled with decisions often made based on a distortion of the risks and benefits of treatments, deriving from a lack of understanding of the science of medicine [27]. This often-dogmatic need for surgery will undoubtedly contribute to the waste and harms of modern medicine and divert resources away from the delivery of effective care. The overall carbon footprint and the impact of climate change and adverse weather events are largely experienced in countries with developing economies; it seems as though this is an acceptable cost in developed nations to deliver care [13,28].

To meet net zero carbon in surgical services, healthcare professionals need to support public health measures to reduce surgical disease, optimise surgical patient pathways and associated processes, and focus on operations with the greatest clinical benefit. Surgeons need to challenge the single-use culture, and shift to reusables across all settings, including operating theatres, emergency departments, and outpatients. Quantifying the environmental impact aids in determining the potential value of mitigation and harm associated with healthcare delivery. Reducing harm should be considered an extension of efforts to improve healthcare, rather than a discrete activity [29]. This change will require leadership, collaboration with supporting services and industries, research evidence, and education [28].

### Limitations

This study’s limitations included a reliance on production emission factors to approximate upstream emissions in the surgical supply chain, as well as the omission of pharmaceutical data and personal/private transportation for staff members. The volumes of biomedical waste were estimated from a representative sample using waste audits rather than having access to all the actual annual waste data for each stream. Of the 200 cases, 20% were directly observed and the rest were extrapolated based on the best estimates. The number of operations performed in the hospital during this period is a six-year average based on figures obtained from the recent annual report, with the assumptions that normal practice resumed in the year 2021 [15]. The estimation of the emissions from operating theatres was based on a limited sample and, thus, entailed a degree of uncertainty. Despite this, the crude estimate was beneficial in helping to recognise the carbon footprint of surgical services. The data does not account for the increase of the disposable of personal protective equipment waste due to COVID-19 infection control measures, in addition to changes in operative case load.

## 5. Conclusions

The challenge to net zero GHG presents an opportunity to catalyse innovation and sustainability in surgery in general, as well as neurosurgery, including how care is delivered, the equipment used, and the surgical methodology. This should, moving forward, improve the quality of healthcare provision to patients and yield potential cost savings. Climate change is currently one of the greatest threats to individual health and society at large. The first steps would be to continue to address and identify the biomedical waste burden of surgical services and to develop strategies to mitigate against this. It would then follow to implement further strategies to reduce emissions through innovation. A vital component of healthcare leadership and environment stewardship should include steps to mitigate against the anthropogenic climate impacts of healthcare activities.

## Figures and Tables

**Table 1:** Annual waste volume and case load by type for a neurosurgical trainee in the UK in a single unit.

	Cases (N)	Total Waste (Kg) Generated	Average (Kg/Case)
Cranial			
Tumour	47	513.33	11.00
Non-tumour	40	320.00	8.00
Average (kg/case)			9.62
Spinal			
Fixation	7	80.00	12.00
Non-fixation	60	540.00	9.00
Average (kg/case)			9.30
Other			
Injections	18	106.67	6.00
Muscle biopsies	4	26.67	6.00
Peripheral nerve	24	195.56	8.00
Average (kg/case)			7.05
Total	200	1782.22	8.91

**Table 2:** Waste volumes secondary calculated greenhouse gas emissions due to neurosurgical consumables per neurosurgical trainee (200 cases operated on within 12 months).

	Waste (Kg/Year *)	CO_2_e (Kg/Year)
Municipal solid waste	643.6	2534.6
Hazardous waste	628.5	1806.3
Reusable textiles	260.3	96.2
Fluid waste	120.3	1.5
Sharps	75.1	342.7
Cytotoxic waste	0.0	0.0
Recycling ^†^	35.8	88.7
Domestic waste	7.7	18.0
Transport ^‡^	10.9	13.4
Total	1782.2	4901.4

CO_2_e = CO_2_ equivalents. * An estimation was made of the emissions of various material waste streams: glass (895 kg CO_2_e per tonne); plastics (3179 kg CO_2_e per tonne); and steel (2708 kg CO_2_e per tonne). Except transport, where the units are km/year. ^†^ Recycling includes cardboard, plastic, and the majority of surgical wrap (polypropylene); the production emission factors used were 1038 kg CO_2_e per tonne for cardboard, 3179 kg CO_2_e per tonne for average plastics, and 3254 kg CO_2_e per tonne for polypropylene; the net emissions with recycling were −240 kg CO_2_e per tonne for cardboard, −282 kg CO_2_e per tonne for average plastics, and 12 kg CO_2_e per tonne for polypropylene. ^‡^ Assumption of 3 km per litre mean fleet fuel efficiency.

## Data Availability

Data are available on request.

## Appendix A

**Table A1:** Page 44 of UHCW Annual report 2018–2019 and page 32 of the UHCW Annual Report 2020/2021—yearly update of waste figures by route of disposal [15,19].

Waste		2015/2016	2016/2017	2017/2018	2018/2019	2019/2020	2020/2021
Recycling	(tonnes)	1832	1904.5	2186.61	1816.57	1274.00	1454.00
	tCO_2_e	36.64	39.99	47.58	38.15	27.72	31.54
Other recovery	(tonnes)	1287	2669.41	1496.43	1509.09	1817.00	1611.00
	tCO_2_e	25.74	56.06	32.56	31.69	39.54	35.06
High temperature disposal	(tonnes)	7	0	0	0	0	0
	tCO_2_e	1.53	0	0	0	0	0
Landfill	(tonnes)	1105	0	0	0	0	0
	tCO_2_e	270.08	0	0	0	0	0
Total Waste (tonnes)		4231	4573.86	3683.04	3325.66	3091.00	3065.00
% Recycled or Re-used		43%	42%	59%	55%	41%	47%
Total Waste tCO_2_e		333.99	96.05	80.14	69.84	67.26	66.69
